# Blood Microbiome Analysis Reveals Biomarkers of Treatment Response in Drug-Naïve Patients with First-Episode Psychosis: A Pilot Study

**DOI:** 10.3390/microorganisms13081935

**Published:** 2025-08-19

**Authors:** Marianthi Logotheti, Thomas Gkekas, Panagiotis C. Agioutantis, Alex Hatzimanolis, Stefania Foteli, Diomi Mamma, Nikolaos C. Stefanis, Fragiskos N. Kolisis, Heleni Loutrari

**Affiliations:** 1 First Department of Critical Care Medicine and Pulmonary Services, Evangelismos Hospital, School of Medicine, National and Kapodistrian University of Athens, 10676 Athens, Greece; mlogotheti@hotmail.com (M.L.); thomasgkekas@mail.ntua.gr (T.G.); 2 Biotechnology Laboratory, School of Chemical Engineering, National Technical University of Athens, 15772 Athens, Greece; panagiout@gmail.com (P.C.A.); dmamma@chemeng.ntua.gr (D.M.); kolisis@chemeng.ntua.gr (F.N.K.); 3 First Department of Psychiatry, Eginition Hospital, School of Medicine, National and Kapodistrian University of Athens, 11528 Athens, Greece; alhatzi@med.uoa.gr (A.H.); stefaniafotelis@gmail.com (S.F.); nistefan@med.uoa.gr (N.C.S.)

**Keywords:** first-episode psychosis, schizophrenia, blood microbiome, dysbiosis, antipsychotics, biomarkers, treatment response, cytokines

## Abstract

Patients with First-Episode Psychosis (FEP) exhibit variable responses to antipsychotic treatment. Emerging evidence suggests that disease-related dysbiosis of gut and oropharyngeal microbiota may lead to the abnormal translocation of microorganisms via the bloodstream. This study aims to explore the blood microbiome to identify candidate biomarkers associated with treatment outcomes in FEP. To address this, blood samples were collected from twenty drug-naïve individuals with FEP, both before and after four weeks of antipsychotic medication. DNA extracted from these samples underwent 16S rRNA gene sequencing and comprehensive bioinformatics analysis. Clinical assessments were based on the Positive and Negative Syndrome Scale and standard remission criteria. Peripheral cytokines (IL1β, TNF-α, IL10) were quantified by immunoassays. Baseline comparisons showed a significantly greater microbiome alpha diversity in remitters, along with differential prevalence in five taxa and 217 metabolic pathways. Post-treatment assessments uncovered a significantly distinct impact of antipsychotics on blood bacterial composition between remission groups, while initial differences on metabolic profiles persisted. Additionally, strong correlations were observed, linking specific taxa abundances to cytokine levels. Conclusively, this pilot study suggests that blood microbiome profiling could provide novel biomarkers for predicting therapeutic response in early psychosis, paving the way for precision medicine interventions.

## 1. Introduction

Schizophrenia (SCZ) and other psychotic disorders rank among the leading global causes of disability-adjusted life years, affecting approximately 1% of the world’s population. SCZ is characterized by psychotic symptoms, negative symptoms (e.g., anhedonia, diminished initiative, social withdrawal), and cognitive impairments. Delayed detection of early phase and prolonged untreated psychosis can significantly impact clinical and functional outcomes. Although substantial advancements have been made in early psychosis research over the past two decades, critical gaps remain in understanding its pathophysiological mechanisms and developing personalized approaches for diagnosis, prevention, and treatment [1,2]. In earlier studies, psychotic disorders have been considered and treated as a unified entity despite significant heterogeneity in symptomatology, leading to considerable variability in patient responses to antipsychotic therapy [3]. Early and effective intervention for First-Episode Psychosis (FEP) is crucial for improving both short- and long-term prognosis. However, reliable biomarkers predicting therapeutic response or resistance remain elusive [4].

Recent research highlights the gut microbiome’s involvement in the pathophysiology of psychiatric disorders, primarily via the microbiota–gut–brain axis, while microbiome-specific biomarkers are being explored to better understand clinical heterogeneity among psychiatric patients [5,6]. Concerning psychotic disorders, most studies rely on cross-sectional microbial comparisons between healthy and affected individuals [7,8,9]. FEP patients exhibit altered gut microbiome structures linked to symptom severity and treatment responsiveness [10,11,12]. Furthermore, exposure of individuals with SCZ to various antipsychotic classes leads to distinct gut microbial signatures influencing treatment outcomes [13] and microbiome alpha diversity [14]. Risperidone has been shown to significantly modify gut bacteria composition in drug-naïve FEP patients, possibly through drug-induced metabolic alterations [15]. Conversely, evidence suggests that gut bacteria may enhance olanzapine bioavailability, though underlying mechanisms remain unclear [16]; potential processes such as biotransformation and bioaccumulation are yet to be elucidated [17]. These findings are in line with the well-acknowledged bidirectional interplay between human gut microbiota and pharmacotherapy, wherein medications modulate microbial composition and function, influencing the treatment outcome, while, in turn, gut bacteria impact drug metabolism, thus affecting bioavailability, tolerability, and efficacy [18,19].

From an immunological perspective, inflammation plays a crucial role in psychotic disorders and antipsychotic response [20,21,22,23,24]. However, the mechanisms underlying its impact on symptom severity and therapeutic outcomes remain poorly understood, while stratification approaches based on cytokine profiles have not proved to be effective thus far [25]. Since gut microbiome dysbiosis in SCZ has been associated with immune dysregulation, and several studies have explored the interplay between pro-inflammatory signaling and microbial composition, probing its contribution to disease pathology [26,27,28].

Beyond the established role of intestinal microbiota, increasing interest has emerged in exploring the structure and functional role of the blood microbiome under both physiological and pathological conditions [29]. The accessibility and minimally invasive collection of blood samples greatly facilitate these investigations. Initially, Nikkari et al. challenged the conventional notion of blood sterility by detecting bacterial DNA in healthy individuals [30]. Subsequent studies confirmed the presence of live microbes or bacterial DNA in both normal and pathological conditions such as diabetes, cardiovascular diseases, asthma, liver fibrosis/cirrhosis, Alzheimer’s disease, and Parkinson’s disease [31,32]. Although the blood microbiome is considered dormant, not related to severe complications (as in sepsis), its interactions with the host immune system may influence homeostasis [29]. The structure of blood microbiota under conditions of disease-related dysbiosis is likely attributed to the aberrant microbial translocation from other niches (gut, oral, skin) as a result of local barrier disruption and increased permeability [33,34,35].

In SCZ, research on the blood microbiome remains scarce, particularly regarding its role in therapeutic response. Transcriptomic analyses have demonstrated increased microbial diversity in SCZ patients compared to healthy controls [36]. Elevated serological biomarkers of bacterial translocation and alterations in microbial-derived metabolites further suggest an immunomodulatory role [26,27,37]. However, blood microbial dynamics throughout treatment remain unexplored.

In this study, we systematically assessed blood microbiota composition and inferred metabolic functions in relation to remission and inflammatory status among drug-naïve FEP patients before and after four weeks of antipsychotic treatment. The proposed study design enabled us to address key questions: first, whether baseline blood microbiota composition harbors prognostic indicators for clinical remission; second, how antipsychotics modulate blood microbial composition and function, and their relevance to treatment outcomes; and third, whether specific microbial components associate with serum cytokine levels, shedding light on interactions between blood microbiota and systemic inflammation in FEP. These findings establish a foundation for future investigations into blood microbiome–host biomarkers that are predictive of antipsychotic response, ultimately guiding personalized therapeutic strategies.

## 2. Materials and Methods

### 2.1. Study Sample and Treatment

The present pilot study included twenty patients diagnosed with FEP. A priori power analysis was conducted to estimate the minimum sample size required to detect significant within-subject changes in clinical outcomes after initiating antipsychotic treatment. Effect size estimates were derived from previously published Positive and Negative Syndrome Scale (PANSS) score data in FEP patients, comparing baseline and four-week follow-up post antipsychotic treatment [38]. Based on these estimates (Cohen’s d = 0.98 for the full sample and d = 1.76 for the patient subgroup achieving remission), a two-tailed Wilcoxon signed-rank test indicated that a minimum of 11 participants overall, and at least 6 in remission, would be required to achieve 80% power at a 5% significance level. Subjects were recruited from the extensive longitudinal cohort of the “Athens First-Episode Psychosis Research Study” which has been previously described in detail [39]. Eligibility criteria mandated the presence of FEP and a confirmed diagnosis of SCZ according to ICD-10 diagnostic standards [40]. Participants were male and either drug-naïve or minimally exposed (≤2 weeks) to antipsychotic medication at the time of enrollment. General exclusion criteria encompassed acute or chronic medical illnesses necessitating anti-inflammatory and/or antibiotic treatment, psychotic disorders secondary to another medical condition or acute intoxication, IQ < 70, developmental disorders, and kinship with an enrolled participant.

Ethical approval was obtained by the Medical Ethics Committee of Eginition Hospital. Every participant provided written informed consent, including detailed information on procedures, risks, and benefits associated with the study.

In accordance with the study design, clinical assessment, blood sample collection, and body mass index (BMI) measurements were conducted at two time points: at baseline (admission) and following four weeks of antipsychotic treatment. Peripheral blood samples were collected from fasting patients via sterile venipuncture using vacuum-sealed EDTA tubes under strict aseptic conditions. All subsequent handling of samples was conducted under a Class II biological safety cabinet to ensure aseptic procedures and minimize cross-contamination. Atypical antipsychotics constituted the mainstay of therapy. Comprehensive demographic, clinical, and medication-related data for each participant are presented in Table 1.

### 2.2. Clinical Measurements

The severity of patients’ symptoms was assessed by the PANSS score [41] at baseline and at four-week follow-up, in order to evaluate antipsychotic treatment efficacy. Remission of SCZ symptoms was assessed based on criteria proposed by Andreasen et al. (2005), with the exception of the time criterion, in accordance with previous studies [42].

**Table 1:** Characteristics of recruited FEP patients and antipsychotic treatment details.

Patient Number	AGE	PANSS		BMI ^c^		Antipsychotics Treatment		Hospitalization	Remission ^f^
		(t_0_) ^a^	(t_1_) ^b^	(t_0_)	(t_1_)	At t_0_ (days ^e^)	t_0_ to t_1_		
1	31	68	46	29.36	28.73		risperidone	YES	YES
2	28	116	76	24.48	28.40		risperidone	YES	NO
3	34	114	73	18.42	18.77		olanzapine, haloperidol	YES	NO
4	21	93	67	21.20	23.60	risperidone (2)	risperidone	YES	NO
5	23	76	54	20.70	22.00		risperidone	YES	NO
6	24	74	38	25.50	27.50		olanzapine, amisulpride	YES	YES
7	28	75	41	25.30	26.50		aripiprazole	YES	YES
8	20	87	59	23.12	25.76	olanzapine (3)	olanzapine	YES	NO
9	28	81	52	24.81	25.46	olanzapine (10)	olanzapine	YES	NO
10	20	79	45	28.40	NA ^d^		olanzapine, amisulpride, aripiprazole	YES	NO
11	31	136	36	23.59	24.62		risperidone	YES	YES
12	21	124	40	30.10	30.10		haloperidol	YES	NO
13	39	161	53	22.40	21.77	haloperidol (1)	risperidone	YES	NO
14	25	180	103	31.80	40.00		risperidone, quietapine	YES	NO
15	35	90	35	23.08	23.66	haloperidol (3)	olanzapine, haloperidol	YES	YES
16	22	110	63	23.78	23.78	haloperidol (2)	olanzapine, haloperidol	YES	NO
17	21	113	55	20.50	20.58		aripiprazole, haloperidol	YES	YES
18	20	75	43	25.24	27.45	risperidone (3)	risperidone, escitalopram	NO	YES
19	24	98	62	20.56	NA		olanzapine	YES	NO
20	24	89	55	27.50	27.60	aripiprazole (13)	aripiprazole	NO	YES
Mean	25.95	101.95	54.80	24.49	25.90	4.50			
SD	5.61	30.27	16.47	3.54	4.65	4.44			

^a^ t_0_: baseline—time of admission. ^b^ t_1_: four weeks from admission. ^c^ BMI in kg/m^2^. ^d^ NA: Not Assigned. ^e^ Length of exposure to antipsychotic at admission. ^f^ Remission assessed at t_1_ according to Andreasen criteria [42], without the time criteria.

### 2.3. Cytokine Measurements

Frozen serum aliquots from FEP participants were obtained at baseline and after four weeks of treatment. Concentrations of IL1β, IL10, and TNF-α were quantified using commercially available human-specific enzyme-linked immunosorbent assay (ELISA) kits, following the manufacturer’s instructions (BioVendor R&D Inc., Brno, Czech Republic). Each sample was analyzed in duplicate. Cytokine concentration values were extrapolated based on optical density measurements derived from standard curves.

### 2.4. Nucleic Acid Extraction

DNA was isolated from whole blood samples using the NucleoSpin^®^ Blood kit (MACHEREY-NAGEL, Düren, Germany) as per the manufacturer’s guidelines, incorporating some modifications to enhance bacterial cell lysis. Briefly, samples were first pre-incubated with 20 mg/mL lysozyme in a 0.8% (*v*/*v*) Triton X-100 solution for one hour at 37 °C. Subsequently, Proteinase K (10% *v*/*v*) and RNase A (2 mg/mL) were added, followed by incubation at ambient temperature for two minutes. All subsequent steps were executed exactly as recommended by the manufacturer. To prevent contaminations, all experimental procedures were conducted within a Class II biological safety cabinet. DNA concentration and purity were assessed spectrophotometrically at 260, 280, and 230 nm using a NanoDrop spectrophotometer (Thermo Scientific, Waltham, MA, USA). DNA aliquots were preserved at −20 °C until further analysis.

### 2.5. 16s rRNA Gene Amplicon Sequencing

The V3–V4 variable regions of the 16S rRNA gene were amplified from DNA extracts using the Illumina Nextera XT DNA Library Prep kit (Illumina, San Diego, CA, USA) with the Forward Primer 341F (5′TCGTCGGCAGCGTCAGATGTGTATAAGAGACAGCCTACGGGNGGCWGCAG), and the Reverse Primer 805R (5′GTCTCGTGGGCTCGGAGATGTGTATAAGAGACAGGACTACHVGGGTATCTAATCC).

Amplicon sequencing was performed at the Greek Genome Center of Biomedical Research Foundation of the Academy of Athens. PCR products (approximately 570 bp) were visualized using microfluidics-based gel electrophoresis on Bioanalyzer 2100 Expert Software B.02.10 (Agilent, Santa Clara, CA, USA) and then purified using AMPure XP (Beckman Coulter, Brea, CA, USA) magnetic bead-based separation. Amplicons were next indexed and sequenced according to the Illumina MiSeq 16S Metagenomic Sequencing Library Preparation Protocol (Illumina, San Diego, CA, USA). Paired-end reads (2 × 250 bp) were generated on an Illumina MiSeqPE250 instrument (Illumina, San Diego, CA, USA). The resulting sequence data were deposited in the Sequence Read Archive and are available under the BioProject PRJNA1181866. Negative controls containing molecular-biology-grade ultrapure water instead of blood (no template controls, NTCs) were processed alongside the patients’ samples from extraction to sequencing.

### 2.6. Quality Control and Taxonomic Assignment

A total of eighty-six paired-end FASTQ files were generated from Illumina sequencing encompassing 20 patient samples obtained at two time points and three NTCs. These files were subsequently demultiplexed and assessed for low-quality reads using FastQC (v0.11.9) [43], before further analysis. The DADA2 (Divisive Amplicon Denoising Algorithm) [44] was employed to infer true biological sequences, which involved primer trimming, the filtering of reads to maintain a mean quality Phred score per read above 26 (max expected error = 0.5), and chimera removal. Extracted Amplicon Sequence Variants (ASVs) were taxonomically classified using the Silva 16S rRNA database [45] via the Naïve Bayesian classifier embedded in the DADA2 (v1.20.0) package within the R environment. A phylogenetic tree of the bacterial community was subsequently constructed using the phangorn R (v2.8.1) package [46]. To mitigate contamination, the decontam (v1.22.0) package in R [47] was applied by integrating bacterial DNA concentrations and the prevalence of ASVs across patient samples and NTCs. Sequence depth adequacy was evaluated using rarefaction analysis, which was performed based on the observed ASVs and total read counts per sample [48].

### 2.7. Measurement of Microbiome Diversity and Differential Taxa Abundance

Microbiome diversity analyses were conducted using the phyloseq (v1.46.0) package in R [49]. Alpha diversity was quantified using multiple metrics (Shannon, Simpson, Observed Taxa, Chao 1) based on the whole group of ASVs. The Wilcoxon signed-rank test was performed to search for statistically significant differences on alpha diversity comparisons. Beta diversity was determined using the Bray–Curtis dissimilarity distance to evaluate compositional differences between groups [50]. Non-metric Multidimensional Scaling (NMDS) [51] was applied to pairwise distance matrices and the k-means clustering algorithm was conducted based on the silhouette method [52] to further examine community structure. For the calculation of Bray–Curtis, the raw counts of bacterial abundance were normalized according to the Trimmed Mean of M-values algorithm [53], in order to mitigate bias associated with library size discrepancies.

Differential abundance analysis was conducted using ANCOM-BC (Analysis of Compositions of Microbiomes with Bias Correction) [54] in R, enabling the identification of taxa exhibiting significant differences in abundance between groups [55]. Taxa present in fewer than 20% of samples were excluded from the analysis. Statistical significance was determined using the False Discovery Rate (FDR) adjusted *p*-values calculated via the Benjamini–Hochberg method [56], with an FDR threshold set at ≤0.05. Group differences were expressed as log_2_ fold-change (FC), with a cutoff value of |log_2_FC| ≥ 1.5.

### 2.8. Functional Analysis

To predict the metabolic pathways associated with the taxonomic composition of the microbiome and estimate their abundances, the PICRUSt2 (Phylogenetic Investigation of Communities by Reconstruction of Unobserved Species) algorithm was implemented based on the entire set of ASVs [57]. PICRUSt2 is a bioinformatics tool that infers the functional potential of microbial communities by aligning 16S rRNA gene sequences to a reference phylogenetic tree, followed by hidden-state prediction, ancestral state reconstruction, and the inference of gene family and metagenomic pathway abundances. Differential abundant pathways across study groups were identified using the ANCOM-BC tool, focusing on pathways that were present in at least 60% of the sample data. Statistical significance was determined using the FDR adjusted *p*-value via the Benjamini–Hochberg correction method, with a threshold of 0.1 while effect size was estimated as a |log_2_FC| with a set cutoff of ≥2.

### 2.9. Quantification of Bacterial Load in DNA Samples

Total bacterial load of DNA samples was measured by quantitative Real-Time PCR (qRT-PCR) amplification of the 16S rRNA gene using universal forward and reverse primers sets [58] F, 5′-ACTCCTACGGGAGGCAGCAGT-3′, and R, 5′-TATTACCGCGGCTGCTGGC-3′. qRT-PCR reactions were conducted in duplicate using a CFX ConnectTM Real-Time PCR System (Bio-Rad Laboratories), with a SYBR green reaction mixture (Boston, MA, USA), 10 nM of each primer, and 1 μL of extracted DNA per reaction. The amplification protocol included an initial denaturation step at 95 °C for 3 min followed by 40 cycles consisting of denaturation at 95 °C for 30 s and annealing/extension at 60 °C for 1 min. A melting curve analysis was performed upon completion. A standard curve for bacterial quantification was constructed using 10-fold serial dilutions of *Escherichia coli* BL21 isolates of known genomic DNA concentrations and colony-forming units plotted against the respective cycle threshold (Ct) value. Total bacterial load in each DNA extract was then determined by interpolating Ct values obtained from the patient samples against this calibration curve.

For relative quantification of *Lactococcus* ASV 35 (sp. *raffinolactis*) into the DNA samples, a similar qRT-PCR reaction protocol was applied using species-specific primers targeting the 16S rRNA gene: F, 5′-CGTTGCATAGAGTGGAAAATTATG-3′, and R, 5′-GTTGAGCCACTGCCTTTTAC-3′. Εqual DNA amounts of test samples were subjected in parallel to 16S rRNA gene amplification by universal primers for internal normalization. The relative abundance of *Lactococcus* ASV 35 was expressed as a percentage of the corresponding total bacterial load.

The Shapiro–Wilk test was applied to assess normality in study parameters. Variables following a normal distribution were analyzed using Student’s *t*-test or paired *t*-tests, whereas non-normally distributed parameters were examined using the Wilcoxon signed-rank test or Mann–Whitney test. Pearson’s correlation coefficients were computed to explore associations between serum cytokine levels, the Shannon index of alpha diversity, and ASV abundance. ASV abundance values were transformed using the centered log-ratio method prior to analysis.

### 2.10. Statistical Analysis

The Shapiro–Wilk test was applied to assess normality in study parameters. Variables following a normal distribution were analyzed using Student’s *t*-test or paired *t*-tests, while non-normally distributed parameters were examined using the Wilcoxon signed-rank test or Mann–Whitney test.

Pearson’s correlation coefficients were computed to explore associations between serum cytokine levels, the Shannon index of alpha diversity, and ASV abundance. ASV abundance values were transformed using the centered log-ratio method prior to analysis. A *p*-value < 0.05 was considered statistically significant while for multiple comparisons, FDR adjusted *p*-values computed using the Benjamini–Hochberg method were applied with a threshold set at 0.05.

## 3. Results

### 3.1. Characteristics and Classification of the Study Sample

The study sample consisted of twenty FEP patients with an average age of 25.95 + 5.61 years. At baseline (t_0_), twelve individuals were drug naïve while eight had minimal prior exposure to antipsychotic treatment, with an average duration of 4.50 + 4.44 days. Following a four-week treatment period with atypical antipsychotics as the primary therapeutic agents (t_1_), a statistically significant improvement was observed in the overall PANSS score (mean values of 101.95 + 30.27 and 54.80 + 16.47 at t_0_ and t_1_, respectively, *p*-value < 0.00001). However, clinical remission assessment at t_1_ based on established criteria [42] divided the cohort into two distinct groups: those who achieved remission (8 patients, 40%, R+, remitters) and those who did not (12 patients, 60%, R−, non-remitters). Descriptive statistical analyses revealed no significant differences in age, BMI, or PANSS scores at admission between remitters and non-remitters (Table 2). As expected, at t_1_, PANSS scores were significantly lower in R+ compared to R−, while the BMI did not differ significantly between the two groups (Table 2). Regarding the overall impact of treatment on BMI, a slight but statistically significant increase was observed (mean values of 24.49 + 3.54 and 25.90 + 4.65 at t_0_ and t_1_, respectively, *p*-value = 0.013).

**Table 2:** Characteristics of remitters (R+) versus non-remitters (R−).

	Descriptive Statistics	Remitters (R+)	Non-Remitters (R−)	*p*-Value (R+ vs. R−)
t_0_				
**AGE**	Median (IQR)	26 (7.75)	23.5 (7)	0.510 ^a^
**PANSS**	Mean (SD)	90 (23.4)	109.91 (32.57)	0.120 ^b^
**BMI**	Mean (SD)	25.01 (2.71)	24.15 (4.08)	0.580 ^b^
t_1_				
**PANSS**	Mean (SD)	43.63 (7.89)	62.25 (16.65)	0.003 ^b^
**BMI**	Mean (SD)	25.83 (2.70)	25.96 (5.92)	0.950 ^b^

The values of continuous variables were presented as mean ± SD in a case of normal distribution or as median ± interquartile range for parameters that did not follow normal distribution. ^a^ Mann–Whitney U test. ^b^ Student’s *t*-test.

### 3.2. A Discrete Microbial Community Inhabits the Blood of FEP Patients

In light of emerging evidence supporting the presence of circulating microorganisms in both health and disease—challenging the traditional view of blood as a sterile environment [32]—we first aimed to confirm the existence of a distinct blood microbial community in samples collected from FEP patients at t_0_ and t_1_. To minimize contamination, a standardized protocol involving strict aseptic techniques was followed during both sample collection and subsequent processing. Furthermore, three independent NTCs (each derived from a pool of 2–3 samples), were included throughout all experimental steps (DNA extraction, PCR amplification, and sequencing). These controls underwent the full downstream workflow, including library preparation, sequencing, and bioinformatic analysis.

Starting with 86 paired-end FASTQ files containing a total of 11.148.151 raw sequencing reads, we applied a validated data-processing pipeline and obtained a mean of 29.930 ± 16.171 high-quality reads per sample, which were assigned to specific ASVs. Next, using the decontam tool, 42 ASVs that were highly prevalent in NTCs but rare in blood samples were excluded from further analysis, resulting in a refined dataset with a mean of 20.825 ± 6409 reads per patient sample. Furthermore, eight samples were found to be overwhelmingly dominated by 21 identical ASVs assigned to the genus *Lacticaseibacillus*. These were considered likely contaminants introduced during sample handling and were excluded from further analysis. After this filtering step, a total number of 1790 ASVs (listed in Supplementary File S1) were identified in blood samples compared to 177 ASVs found in NTCs. Taxonomic classification revealed the presence of 17 phyla, 33 classes, 76 orders, 125 families, and 226 genera. Rarefaction curves confirmed that all samples had a sufficient sequencing depth to reach saturation in ASV assignment (Supplementary Figure S1). ASVs corresponding to bacterial genera considered to be “unusual” for the human microbiome were not excluded a priori but were rather considered with caution in the analysis [59].

**Figure 1:**
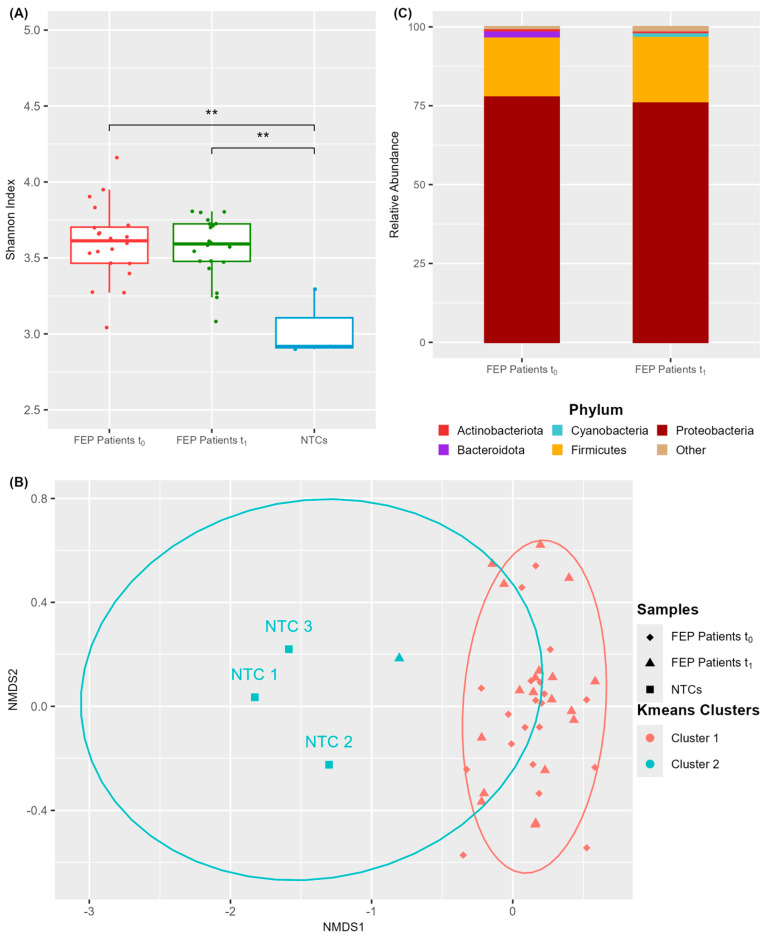
Characterization of the blood microbiome in FEP patients before (t_0_) and after (t_1_) antipsychotic treatment. (**A**,**B**) Comparison of microbiome diversity metrics in FEP patients versus NTCs. Each dot represents an individual sample. (**A**) Shannon index of alpha diversity. (**B**) NMDS ordination of Bray–Curtis dissimilarities and k-means clustering. (**C**) Taxonomic composition of blood microbiome at the phylum level in FEP patients, expressed as mean relative abundance. Phyla with <1% relative abundance were assigned as “Other.”; ** *p*-value < 0.01 (Wilcoxon test).

To further assess the specificity of the blood microbiome, we compared alpha diversity indices (Shannon, Simpson, Observed Taxa, Chao 1) and beta diversity metrics, based on defined ASVs, between patient samples and NTCs (Figure 1 and Supplementary Figure S2A). As shown in Figure 1A, the median intra-individual Shannon alpha diversity index was significantly higher (Wilcoxon test, *p*-value  <  0.005) in samples of FEP patients compared to NTCs at both time points, i.e., before (t_0_) and after treatment (t_1_). No significant difference was found in the median Shannon index between t_0_ and t_1_ within the patient group. Importantly, inter-individual beta diversity analysis (NMDS ordination of Bray–Curtis distances) revealed marked dissimilarity between NTCs and patient samples at both time points (Figure 1B), which was further supported by distinct k-means clustering separating NTCs from patients samples. Consistently, quantification of the total bacterial load of samples by qRT-PCR targeting the 16S rRNA gene demonstrated significantly higher bacterial density (*p*-value = 0.00005) in FEP patient blood samples (mean value: 2.4 × 10^5^ ± 1.2 × 10^5^ CFU/mL) compared to NTCs (mean value: 0.007 × 10^5^ ± 0.001 × 10^5^ CFU/mL). Collectively, these findings strongly support the existence of a distinct blood microbiome in FEP patients.

Finally, to characterize the taxonomic composition of the FEP patient blood microbiota, we calculated the relative abundances of identified taxa (Figure 1C). Four phyla predominated, with no significant differences between time points (values refer to t_0_): Proteobacteria (77.7%), Firmicutes (18.6%), Bacteroidota (1.9%), and Actinobacteria (1.2%).

### 3.3. The Blood Microbiome Composition of FEP Patients Is Related to Treatment Outcome

Given that clinical evaluation of FEP patients after four weeks of antipsychotic therapy classified them in R+ and R− groups, we next investigated potential connections between microbial composition and remission outcome.

#### 3.3.1. A Significantly Distinct Blood Microbiome Structure Characterizes R+ Versus R− at Baseline

**Figure 2:**
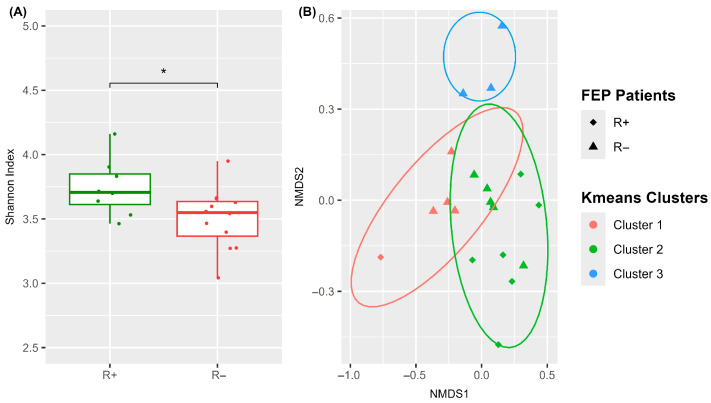
Differences in blood microbiome diversity metrics between remitters (R+) and non-remitters (R−) at baseline. (**A**) Shannon index of alpha diversity. (**B**) NMDS ordination of Bray–Curtis dissimilarities and k-means clustering; * *p*-value < 0.05 (Wilcoxon test).

We first compared the blood microbiome characteristics of R+ and R− patients at baseline (t_0_) to identify differences that were potentially predictive of treatment response (Figure 2 and Supplementary Figure S2B). As shown in Figure 2A, the blood microbiome of R+ patients exhibited a significantly higher Shannon alpha diversity index compared to R− patients (Wilcoxon test, *p*-value = 0.005), indicating that a richer and more diverse microbial community in the blood of drug-naïve FEP individuals may be associated with a more favorable therapeutic response. Analysis of median Bray–Curtis distances between the two groups indicated moderate dissimilarity (median value: 0.44). NMDS ordination of Bray–Curtis distances followed by k-means clustering grouped all samples into three distinct clusters (Figure 2B). Notably, R^+^ samples were predominantly clustered in group 2, whereas R− samples were dispersed across all three clusters, reflecting greater microbiome heterogeneity within the non-responder group.

To further explore specific bacterial taxa that are potentially linked to treatment outcome, we performed differential abundance analysis between R+ and R− patients at baseline. A total of five genera (six ASVs), belonging to the Proteobacteria and Firmicutes phyla, were found to differ significantly in prevalence (FDR < 0.05, |log_2_FC| ≥ 1.5) (Table 3, t_0_: R+ vs. R−). Three genera were more abundant in R+ patients, namely *Lactococcus* (ASV 35, log_2_FC = 4.5), *Enhydrobacter* (ASV 50, log_2_FC = 2.5), and *Esherichia schighella* (ASV 44, log_2_FC = 2.5). In contrast, *Staphylococcus* (ASV 46, log_2_FC = −1.9; ASV 105, log_2_FC = −1.5) and *Acinetobacter* (ASV 106, log_2_FC = −1.7), were less prevalent in R+ patients.

**Table 3:** Differential taxa abundance analysis of bacterial taxa between remitted and non-remitted FEP patient groups and treatment-related states (FDR < 0.05).

Phylum	Genus	ASV	log_2_FC ^a^			
			t_0_ ^b^: R+ ^c^ vs. R− ^d^	t_1_ ^e^: R+ vs. R−	R+: t_1_ vs. t_0_	R−: t_1_ vs. t_0_
Proteobacteria	*Acinetobacter*	ASV 59		−2.1	−2.4	
		ASV 62			3.0	
		ASV 106	−1.7			−1.7
	*Aeromonas*	ASV 77			−2.5	
	*Aliidiomarina*	ASV 57			2.8	
	*Caulobacter*	ASV 30			−2.9	
	*Enhydrobacter*	ASV 50	2.5			
	*Escherichia-Shigella*	ASV 44	2.5			1.9
	*Methylobacterium-Methylorubrum*	ASV 45		2.2		
		ASV 68			−1.8	
		ASV 81			−2.3	
	*Morganella*	ASV 89		2.1		
	*Paracoccus*	ASV 94		−1.9		2.6
Firmicutes	*Anoxybacillus*	ASV 87		−1.7		
	*Bacillus*	ASV 39			2.4	
	*Lactococcus*	ASV 35	4.5			
		ASV 69				−2.7
	*Staphylococcus*	ASV 46	−1.9	−1.8		
		ASV 105	−1.5			−2.2
	*Streptococcus*	ASV 58		−2.9	−1.9	
Bacteroidota	*Cloacibacterium*	ASV 47			−1.5	
		ASV 84			−1.7	

^a^ log_2_FC: Fold change in log_2_ value; threshold was set at 1.5 for the |log_2_FC|. ^b^ t_0_: baseline—time of admission. ^c^ R+: Remitters. ^d^ R−: Non-Remitters. ^e^ t_1_: four weeks from admission.

#### 3.3.2. Antipsychotics Differentially Impact the Blood Microbiome Structure in R+ Versus R− FEP Patients

**Figure 3:**
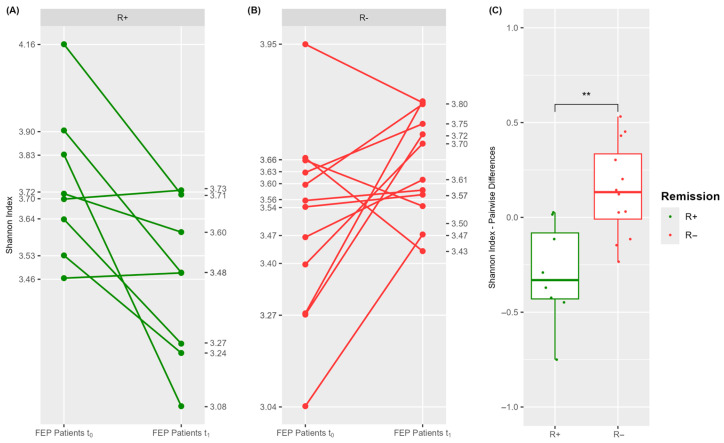
Impact of antipsychotic treatment on alpha diversity (Shannon index) of the blood microbiome in FEP patients. (**A**,**B**) Spaghetti plots showing individual trajectories from t_0_ to t_1_ for R+ (**A**) and R− (**B**) participants. (**C**) Box plots of pairwise differences in alpha diversity between t_1_ and t_0_; ** *p*-value < 0.01 (Wilcoxon test).

In light of the above findings, we next investigated whether antipsychotic medication exerts differential effects on the blood microbiome composition of R+ versus R− patients. To address this, we visualized individual changes in alpha diversity scores before and after treatment using spaghetti plots (Figure 3A,B) and calculated pairwise differences within each group (Figure 3C and Supplementary Figure S2C).

Notably, alpha diversity was substantially reduced in most R+ patients, whereas it increased in the majority of R− patients, following treatment. This suggests that pharmacotherapy induced a significantly divergent effect on patient blood microbiome richness depending on treatment response.

To further explore post-treatment dissimilarities and identify drug-targeted taxa, we performed differential abundance analyses between R+ and R− patients at t_1_, as well as within each group at t_1_ versus t_0_. The results are summarized in Table 3 (FDR < 0.05, |log_2_FC| ≥ 1.5). In total seven genera were differentially abundant between R+ and R− patients after treatment (Table 3, t_1_: R+ vs. R−), with five showing reduced and two showing increased abundance in R+. Specifically, *Streptococcus* (ASV 58, log_2_FC = −2.9), *Acinetobacter* (ASV 59, log_2_FC = −2.1, *Paracoccus* (ASV 94, log_2_FC = −1.9), *Staphylococcus* (ASV 46, log_2_FC = −1.8), and *Anoxybacillus* (ASV 87, log_2_FC = −1.7) were less abundant in R+, while *Methylobacterium-Methylorubrum* (ASV 45, log_2_FC = 2.2) and *Morganella* (ASV 89, log_2_FC = 2.1) were enriched.

The most extensive changes were observed within the R+ group when comparing t_1_ to t_0_. As demonstrated in Table 3 (R+: t_1_ vs. t_0_), treatment significantly altered the abundance of eleven ASVs (eight decreased and three increased), belonging to eight genera. Notable reductions were observed for *Methylobacterium-Methylorubrum* (ASV 81, log_2_FC = −2.3 and ASV 68, log_2_FC = −1.8), *Streptococcus* (ASV 58, log_2_FC = −1.9), and *Acinetobacter* (ASV 59, log_2_FC = −2.4), while a distinct *Acinetobacter* ASV (ASV 62, log_2_FC = 3.0) showed increased abundance. *Lactococcus* levels also decreased post-treatment, though to a lesser extent (log_2_FC = −1.2).

In the R− group, drug exposure significantly altered the abundance of five ASVs/genera (Table 3, R−: t_1_: vs. t_0_). Decreases were observed in *Lactococcus* (ASV 69, log_2_FC = −2.7), *Staphylococcus* (ASV 105, log_2_FC = −2.2), and *Acinetobacter* (ASV 106, log_2_FC = −1.7), while increases were noted for *Paracoccus* (ASV 94, log_2_FC = 2.6) and *Esherichia-Schighella* (ASV 44, log_2_FC = 1.9).

These findings were further validated by qRT-PCR in representative patients, particularly for *Lactococcus*, which showed prominent differential abundance in both basal and post-treatment comparisons (Supplementary Figure S3).

### 3.4. Functional Diversity of Blood Microbiome in R+ Versus R− Patients

**Figure 4:**
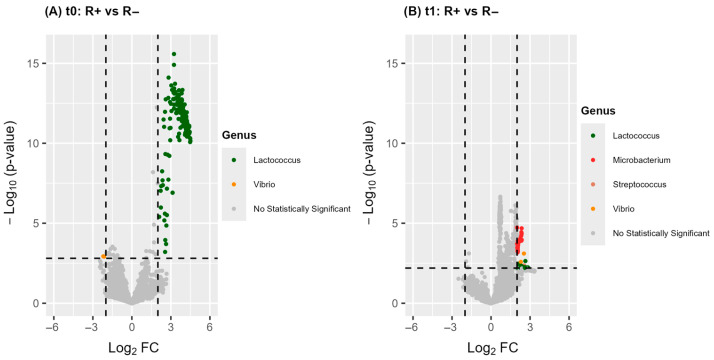
Differential abundance analysis of predicted metabolic functions of the blood microbiota in FEP patients based on the comparisons of R+ vs. R− at t_0_ (**A**) and at t_1_ (**B**). Colored dots represent significantly modified pathways and their connection to a specific bacterial genus labeled by the same color. Gray dots indicate not significantly differentiated metabolic pathways. Statistical threshold value for the FDR adjusted *p*-value was 0.1. The threshold value for effect size was |log_2_FC| ≥ 2.

**Figure 5:**
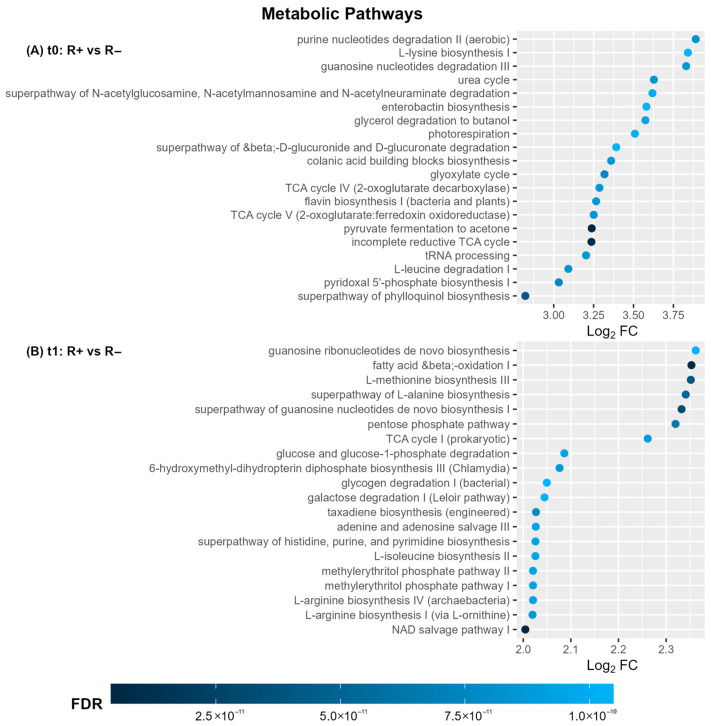
Top twenty differential abundant metabolic pathways in the blood microbiota of FEP patients as ranked by FDR value in blood microbiota based on the comparison of R+ vs. R− at t_0_ (**A**) and t_1_ (**B**). Statistical threshold value for the FDR adjusted *p*-value was 0.1. The threshold value for effect size was |log_2_FC| ≥ 2.

We next explored potential differences in the functional profile of the blood microbiome between patient remission groups, both before and after treatment. This investigation combined data from PICRUSt2 with the differential abundance analysis of predicted metabolic pathways. The complete list of pathways is provided in Supplementary File S2 (FDR < 0.1, |log_2_FC| ≥ 2.0). Figure 4 depicts the corresponding volcano plots of differential abundant pathways while Figure 5 highlights the top 20 pathways ranked by FDR value. For each comparison, the bacteria genera contributing to the observed differences are also indicated.

In total, 217 and 94 pathways showed significantly different abundances between R+ and R− patients, before and after treatment, respectively. Notably, the most prominent differences at both time points involved the upregulation of functional categories related to purine metabolism, amino acid and branched-chain amino acid (BCAA) metabolism, carbohydrate metabolism, cofactor and vitamin biosynthesis, and energy production pathways (Figure 5A,B). At baseline, the predicted metabolic differences were almost exclusively attributed to *Lactococcus* (ASV 35, sp. *raffinolactis*) whereas at t_1_, a broader range of bacterial taxa contributed to the observed functional divergence (Figure 4A,B). Overall, these findings highlight a substantially distinct functional profile of the blood microbiome between R+ and R− patients and further indicate that antipsychotic treatment may exert a differential effect on microbiome metabolism depending on the remission group.

### 3.5. Integration of Blood Microbiome and Peripheral Cytokine Data

Given that immune dysfunction has been proposed as a predictive biomarker of antipsychotic response in individuals with FEP [4], we investigated whether features of the blood microbiota are associated with circulating cytokine levels. To this end, we measured serum concentrations of three representative cytokines, namely, TNF-α, IL1β, and IL10, at t_0_ and t_1_ (Supplementary Table S1). As shown in Supplementary Figure S4, we observed a common trend toward reduced levels of the pro-inflammatory cytokines TNF-α and IL-1β following treatment. We next performed Pearson correlation analysis to explore potential links between microbiome alpha diversity (Shannon index) and cytokine levels, both before and after treatment. As shown in Supplementary Table S2, only weak, not significant correlations were observed. We further examined whether treatment-induced changes in microbiome diversity were associated with changes in cytokine expression by analyzing pairwise differences between t_1_ and t_0_. A significant negative correlation was identified between changes in Shannon index and IL10 levels (Pearson Ro = −0.59, *p*-value = 0.03, Supplementary Table S2), indicating potential cross-talk between anti-inflammatory pathways and the microbiome in response to treatment.

Finally, to uncover specific blood microorganisms related to host inflammation in FEP, we examined potential associations between the normalized counts of taxa listed in Table 3 and cytokine levels across all participants. The analysis (Table 4) identified five genera (comprising seven distinct ASVs) that exhibited a strong trend of correlation—positive or negative—with at least one cytokine at either time point (t_0_ or t_1_). Specifically, at baseline, four cytokine-associated genera were detected: *Cloacibacterium* (ASV 47, positively correlated with TNF-α), *Escherichia-Shigella* (ASV 44, negatively correlated with IL-10), *Methylobacterium-Methylorubrum* (ASV 45, negatively correlated with TNF-α; ASV 68, negatively correlated with IL-10), and *Paracoccus* (ASV 94, positively correlated with IL-1β).

**Table 4:** Pearson correlation analysis between specific blood microbiome taxa and serum cytokine levels in FEP patients.

Time Point	Genus	ASV	Cytokine	Pearson Ro	*p*-Value	FDR
t_0_ ^a^	*Cloacibacterium*	ASV 47	TNF-α	0.60	0.038	0.418
	*Escherichia-Shigella*	ASV 44	IL10	−0.58	0.038	0.514
	*Methylobacterium-Methylorubrum*	ASV 45	TNF-α	−0.63	0.028	0.418
	*Methylobacterium-Methylorubrum*	ASV 68	IL10	−0.56	0.047	0.514
	*Paracoccus*	ASV 94	IL1β	0.57	0.042	0.928
t_1_ ^b^	Acinetobacter	ASV 106	IL10	0.80	0.001	0.023
	*Escherichia-Shigella*	ASV 44	IL1β	−0.60	0.030	0.515
	*Methylobacterium-Methylorubrum*	ASV 81	TNF-α	−0.58	0.036	0.558
	*Methylobacterium-Methylorubrum*	ASV 68	IL10	0.67	0.012	0.133

^a^ t_0_: baseline—time of admission. ^b^ t_1_: four weeks from admission.

Furthermore, following treatment, a very strong and statistically significant positive correlation was observed between *Acinetobacter* (ASV 106) and IL10 (Pearson Ro = 0.80, *p* = 0.001, FDR = 0.023). Additional post-treatment associations included a negative correlation between *Escherichia-Shigella* (ASV 44) and IL-1β, a positive correlation between *Methylobacterium-Methylorubrum* (ASV 68) and IL-10, and a negative correlation between *Methylobacterium-Methylorubrum* (ASV 81) and TNF-α. Collectively, these findings support the existence of dynamic interactions between components of the blood microbiome and the host’s systemic inflammatory response in FEP.

## 4. Discussion

Alterations in the blood microbiota of patients with SCZ compared to healthy individuals have been previously reported [36] highlighting the potential to explore host–microbiome interactions using an accessible biological material. Such investigations may offer insights into disease prognosis and improve therapeutic management. In this context, we undertook a pilot prospective study that enrolled twenty drug-naïve FEP patients and proceeded to a dynamic analysis of the blood microbiome landscape before and after four weeks of treatment. Our aim was to evaluate potential treatment effects and identify microbiome dissimilarities between patients with different remission statuses. Additionally, we sought to examine associations between specific blood microbiome features and peripheral levels of IL1β, TNF-α, and IL10—cytokines implicated in systemic inflammation and responsiveness to antipsychotic therapy in SCZ patients [23].

Given the inherently low microbial biomass of peripheral blood, we implemented rigorous contamination controls. These included (a) stringent aseptic protocols during blood collection and further handling, (b) the inclusion of NTCs as experimental negative controls, and (c) bioinformatics tools to identify and eliminate potential contaminants [47]. The presence of a distinct blood microbiome in FEP patients, as opposed to NTCs, was confirmed by NGS analysis (Figure 1) and further corroborated by qRT-PCR quantification of total bacterial load (Supplementary Figure S3). Some ASVs were taxonomically assigned to atypical groups not commonly associated with the human microbiome. These were presumed to reflect either extremely rare opportunistic taxa or environmental contaminants, and their relevance was therefore cautiously interpreted [59].

The blood microbiome in FEP patients was predominantly composed of Proteobacteria, followed by Firmicutes, Bacteroidota, and Actinobacteria (Figure 1C). Earlier 16S rRNA-based metagenomic findings in healthy individuals have shown the same predominant phyla [31,34]. The main distinction observed was an elevated Firmicutes-to-Actinobacteria ratio in FEP compared to healthy subjects. Similar compositional profiles have been reported in transcriptome-based studies in SCZ, with the exception of Cyanobacteria, which were exceedingly rare in our samples (<1%) [36]. Interestingly, the oral microbiome of SCZ patients has shown a comparable phylum-level distribution [60]. A large-scale study on the blood microbiome in healthy individuals proposed that blood harbors no stable core microbiome but instead reflects a transient and sporadic translocation of commensals from various body sites [61]. This hypothesis aligns with the concept of atopobiosis, describing the sporadic appearance of microbes outside their typical niches, without stable colonization [29]. It is thus rational to assume that SCZ-associated dysbiosis in the oral [60] and gut [8] microbiomes may alter the frequency and characteristics of microbial translocation events, leading to blood microbial profiles that are reflective of compromised barrier integrity and microbial dynamics at distant body sites. Mechanistically, the gut and oral mucosa are considered primary microbial reservoirs, where barrier disruptions—such as increased intestinal permeability (“leaky gut”) or breaches in oral epithelial defenses—facilitate microbial entry into circulation [32]. Moreover, translocation can occur via specialized cellular pathways, including dendritic cell-mediated transport and antigen sampling by M cells within gut-associated lymphoid tissues [29,34]. Other potential sources, including the skin and even maternal transmission, have been hypothesized, but their contributions to the circulating microbiome in health and disease are yet to be fully elucidated [34].

Further investigation revealed that the blood microbiome of patients who responded to treatment (R+) differed significantly—compositionally (Figure 2, Table 3) as well as functionally (Figure 4A,B and Figure 5A,B)—from that of non-responders (R−), at both baseline and post-treatment. At baseline, drug-naïve R+ patients exhibited greater Shannon alpha diversity, indicating increased microbial complexity, along with more homogeneous inter-individual microbiota profiles. They also displayed significant differences in the relative abundance of five genera and 217 predicted metabolic pathways (Figure 2, Figure 4, and Figure 5; Table 3). Notably, PICRUSt2 analysis revealed an enhanced metabolic profile in R+ patients, characterized by elevated biosynthetic and energy-producing pathways. At the taxonomic level, the most outstanding difference in participants who achieved remission was the prevalence of *Lactococcus* (ASV 35, *L. raffinolactis*) which was also the principal contributor to the enriched metabolic activity observed in this group. These results support its potential role as a prognostic biomarker of remission in drug-naïve FEP patients, that possibly modulates host response through the synthesis of essential microbial metabolites such as cofactors/vitamins, purines, and amino acids/BCAAs; Figure 5A). This hypothesis aligns with previous evidence supporting the health-promoting properties of lactic acid-producing bacteria, such as Lactococcus and Lactobacillus spp., in maintaining gut microbiota balance and regulating host immunity and behavior [62,63]. *Lactococcus lactis* has been extensively studied as a mucosal delivery vehicle for therapeutic agents [64], while *Lactococcus raffinolactis* has recently been assessed for its in vitro probiotic activity and anti-neuroinflammatory effects on human oligodendrocytes [65]. Moreover, SCZ patients with significantly higher PANSS scores have been shown to possess significantly lower brain lactate concentrations compared to those with lower PANSS scores [66]. In addition, a recent meta-analysis has shown that certain *Lactobacillus* strains may exert neuroprotective or anti-inflammatory actions when administered as probiotics, supporting their potential therapeutic role in schizophrenia [67]. In contrast to the aforementioned findings, several reports have presented evidence of a significant increase in *Lactobacillus* species in individuals with SCZ compared to healthy controls [10,68]. For instance, elevated levels of gut Lactobacilli have been correlated with greater severity of clinical symptoms in FEP patients [10]. Furthermore, a dysbiotic “imbalance” in *Lactobacilli* has also been reported in the oral microbiome of SCZ patients, alongside the detection of *Lactobacillus phage phiadh* which has been implicated in altering the microbiome ecology in host bacteria [68]. These contradictory observations likely reflect a combination of factors including strain-specific microbial effects, host-related clinical heterogeneity, and differences in the context of abundance (endogenous versus controlled probiotic supplementation), study design (cross-sectional versus interventional clinical trials) and methodology. Taken together, these findings underscore the complexity of host–microbiota interactions in SCZ and highlight the need for longitudinal, strain-specific studies that integrate clinical variables and mechanistic insights to clarify the prognostic and therapeutic potential of lactic acid-producing bacteria and their metabolites in disease pathophysiology.

With respect to antipsychotic treatment, prior studies have demonstrated their antimicrobial properties and differential effects on gut microbiome diversity, taxonomy, and function, depending on the clinical outcome of remission [14,19]. Consistent with these findings, we observed that post-treatment changes in blood microbiome alpha diversity, as assessed by the Shannon index, showed a significant decrease in R+ patients, whereas R− patients exhibited a minor, non-significant increase. Furthermore, differential abundance analysis between t_0_ and t_1_ revealed 11 altered ASVs in R+ patients (8 decreased, 3 increased), compared to 5 ASVs in R− patients (3 decreased, 2 increased).

At the functional level, antipsychotic therapy disrupted microbial metabolic activities (Figure 4A,B); however, the microbiome of R+ patients appeared to be more resilient to this effect, maintaining a profile that is enriched in metabolic pathways related to amino acid/BCAA biosynthesis, fatty acid metabolism, purine synthesis, and energy production (Figure 5A,B). Many of these metabolites have been implicated in the regulation of neurotransmitter production, thereby influencing clinical symptoms and therapeutic response in psychiatric disorders [69]. Notably, the biosynthesis of aromatic amino acids—tryptophan, tyrosine, and phenylalanine—was increased in the R+ group relative to R− (Supplementary File S2). These amino acids serve as precursors for serotonin, dopamine, and norepinephrine, all of which are centrally involved in SCZ pathophysiology [2,69,70]. Low circulating levels of tyrosine and tryptophan are associated with the impaired synthesis of these neurotransmitters and the emergence of psychotic symptoms. Moreover, tryptophan-derived metabolites have also been linked to treatment responsiveness in early psychosis [24,71]. We also identified R+ versus R− differences in microbiota-related metabolic pathways involving BCAA, such as valine, leucine, and isoleucine (Supplementary File S2), metabolism. Previous studies have reported elevated BCAA-related pathways and higher BCAA concentrations in the blood and cerebrospinal fluid of SCZ patients compared to healthy controls [72,73,74]. Because BCAAs share transport mechanisms across the blood–brain barrier with aromatic amino acids, any fluctuations in circulating BCAA levels may inversely impact brain concentrations of tyrosine, tryptophan, and phenylalanine. This, in turn, could alter the synthesis and release of key neurotransmitters [75]. An additional finding of interest was the persistent enrichment of microbial pathways associated with acetate metabolism in R+ versus R− patients at both time points. Short-chain fatty acids, including acetate, have been shown to possess immunomodulatory properties within the gut microbiome [76], and recently Ju et al. have highlighted their role in modulating central nervous system function [77]. Acetate, in particular, serves as a metabolic precursor for the synthesis of glutamate and γ-aminobutyric acid [78]. Reduced glutamate levels have been reported in FEP patients, while glutamate antagonists for the N-methyl-D-aspartate receptor, such as kynurenic acid, have also been associated with the pathogenesis of SCZ and FEP [24,79]. Furthermore, decreased levels of γ-aminobutyric acid amounts are linked to psychiatric conditions including anxiety and mood disorders [80]. Taken together, these results support the hypothesis that differences in blood microbiota composition and metabolic potential between R+ and R− patients may lead to distinct profiles of circulating microbial-derived metabolites—many of which serve as precursors to key neurotransmitters implicated in SCZ. Such divergences may ultimately contribute to the observed heterogeneity in therapeutic response following antipsychotic treatment in FEP.

Beyond antipsychotics, other interventions such as antibiotics and probiotics have also been implicated in modulating the microbiome and influencing psychosis outcomes. Prior antibiotic exposure has been associated with increased SCZ risk, likely through microbiome disruption, while certain antibiotics have been linked to neuropsychiatric side effects [81]. Notably, minocycline has shown promise in reducing negative symptoms, whereas fluoroquinolones have been associated with adverse effects [82]. These observations further justify our exclusion criteria regarding antibiotic treatments, which could confound microbiome-related outcomes. Conversely, probiotics—particularly *Lactobacillus* and *Bifidobacterium* strains—have demonstrated preliminary benefits in SCZ, including reduced inflammation and improved PANSS scores [83,84,85]. A recent meta-analysis supports these findings, highlighting mechanisms such as immunomodulation and neurotransmitter regulation [67].

An expanding body of evidence implicates the dysregulation of peripheral cytokines in neuroinflammatory processes that contribute to cognitive and neuroanatomical alterations in SCZ [23,86]. Elevated levels of IL-1β and TNF-α in SCZ patients have been associated with increased severity of psychotic symptoms [86,87], while IL10 has been linked to a mitigating effect on negative symptoms in FEP patients [88]. Our results demonstrated a post-treatment reduction trend in peripheral pro-inflammatory cytokines TNF-α and IL-1β (Supplementary Figure S4), aligning with previous observations in both SCZ and FEP populations [24,86,89].

Given the well-established association between immune dysfunction, gut microbiota dysbiosis, and compromised intestinal barrier integrity [37,86], we explored potential relationships between blood microbiome and host circulating cytokines—an area that remains largely uncharted. Initial correlation analysis between microbiota alpha diversity (Shannon index) and cytokine levels revealed a significant inverse association between treatment-induced changes in microbial diversity and IL-10 levels (Pearson’s R = –0.59, *p* = 0.03). This observation suggests a potential systemic host–microbiome interaction in response to antipsychotic treatment.

Subsequent analysis assessing correlations between specific taxa (22 ASVs listed in Table 3) and cytokine levels identified nine strong associations (Table 4). Among these, a particularly notable finding was the robust post-treatment positive correlation between IL-10 and *Acinetobacter* (ASV106; R = 0.80, *p* = 0.001, FDR = 0.023), suggesting a potential immunomodulatory role for this taxon following pharmacological intervention. The reduced abundance of *Acinetobacter* in R+ compared to R− patients at baseline, and in R− following treatment (Table 3), further supports its relevance to clinical status. While *Acinetobacter* species are recognized as opportunistic pathogens colonizing various human niches (e.g., skin, mucosa, blood [90]), their abundance has also been positively associated with IL-10 in the placental microbiota of women with gestational diabetes [91]. Furthermore, detection of *Acinetobacter* in the bloodstream of type 2 diabetes patients has been linked to elevated circulating cytokine levels, indicating a potential capacity to elicit broad inflammatory responses [92].

Two additional genera, *Methylobacterium-Methylorubrum* (ASV 68) and *Escherichia-Shigella* (ASV 44), demonstrated negative correlations with IL10 at baseline (Table 4), indicating possible pro-inflammatory roles in FEP pathophysiology. Notably, *Methylobacterium–Methylorubrum* was significantly reduced in R+ patients, while *Escherichia–Shigella* significantly increased in R− patients, in response to treatment (Table 3), thus suggesting the potential implication of these taxa to the heterogeneity of therapeutic outcomes. *Methylobacterium* has previously been identified as an opportunistic pathogen associated with increased levels of TNF-α and IL-1β in liver cirrhosis—a disorder characterized by dysbiosis, impaired intestinal function, and microbial translocation. In that context, Methylobacterium was found to produce methanol-related metabolites linked to systemic inflammation [93]. Likewise, the relationship between *Escherichia–Shigella* and pro-inflammatory cytokine expression has been established in multiple studies [94,95].

Another compelling observation was the positive baseline correlation between TNF-α and *Cloacibacterium* (ASV47), suggesting that this genus may also act as an opportunistic circulating pathogen with a pro-inflammatory capacity. The post-treatment decline in *Cloacibacterium* abundance in R+ patients (Table 3) further supports its possible association with clinical remission. Consistent with this, *Cloacibacterium* was found in elevated levels in the bloodstream of Parkinson’s disease patients—correlating positively with disease duration—thereby implicating it as a potential biomarker for neuroinflammation [96]. Additionally, *Paracoccus*—a microorganism reported to be unexpectedly abundant in the gut microbiota of patients with active *Vibrio cholerae* infection and implicated in the pathogen’s virulence [97]—was found to exhibit a positive correlation with the pro-inflammatory cytokine IL-1β at baseline. Notably, following antipsychotic treatment, *Paracoccus* abundance increased in R− patients, while it decreased in R+ compared to R− (Table 3), suggesting a possible association between its modulation and treatment response in FEP.

A general observation arising from this analysis is that the majority of taxa identified as pro-inflammatory, based on their correlation with serum cytokine levels, are Gram-negative bacteria. In support, prior studies have reported elevated circulating levels of lipopolysaccharide-binding protein, soluble CD14, and antibodies to Gram-negative bacteria in SCZ patients relative to healthy controls. These findings are indicative of increased intestinal permeability and systemic exposure to Gram-negative bacterial components in SCZ [23]. Furthermore, lipopolysaccharides derived from Gram-negative bacteria have been shown to activate macrophages, triggering the release of pro-inflammatory cytokines and potentially contributing to a “cytokine storm” effect [93]. Collectively, these results provide novel evidence linking specific blood-resident bacterial taxa to systemic inflammation and therapeutic outcomes in FEP.

Although our correlation analysis identified some strong associations between specific microbial taxa and peripheral cytokine levels, we acknowledge that more advanced network-based approaches, such as SparCC, could offer a deeper understanding of microbiota–host covariation patterns. However, given the limited sample size of this pilot study, we opted for more conservative correlation methods (e.g., Pearson) to reduce the risk of spurious associations. SparCC and similar compositional data network tools typically require larger cohorts to yield robust and interpretable results [98]. Future studies with expanded sample sizes will allow for the implementation of such methods, potentially uncovering more complex interaction networks that underline treatment response in early psychosis.

This pilot study is not without limitations. We acknowledge the brief follow-up period and the relatively small sample size as constraints. However, the use of strict patient inclusion criteria and stratified analysis by remission status aimed to enhance internal validity. Furthermore, the exclusive inclusion of male patients may limit generalizability and preclude conclusions regarding sex-based differences. In fact, emerging evidence indicates that sex significantly influences gut microbiota composition through hormonal and immune mechanisms, introducing the concept of the “microgenderome” [99]. These sex-related microbiome differences have also been associated with variations in SCZ symptomatology, disease progression, and treatment-related metabolic effects [100]. Consequently, the present study’s male-only sample restricts the extrapolation of findings to female populations. Longitudinal investigations in larger, sex-diverse cohorts—with repeated blood sampling beyond the initial four-week period—are essential to validate and expand upon these findings. Additionally, unmeasured confounders such as smoking status, alcohol consumption, and dietary patterns may have influenced our results. Methodologically, as the bacterial community profiles were derived from 16S rRNA amplicon sequencing, our analysis was limited to bacterial taxa, excluding other microbial domains (e.g., viruses, fungi). Moreover, a general caveat of all DNA-based approaches is the inability to distinguish viable from non-viable organisms, as microbial DNA can persist post-mortem. Recent findings suggest that exogenous microbial DNA may activate innate immune pathways such as cGAS-STING and induce cytokine expression independently of microbial viability [101,102]. This raises the possibility that some of the observed associations between microbial taxa and cytokine levels may reflect immunogenic effects of circulating DNA rather than host interaction with active blood microorganisms. Future studies incorporating culture-based assays, viability PCR, or RNA sequencing-based metagenomics will be essential to clarify this distinction [103].

## 5. Conclusions

This study presents a pilot investigation into blood-associated bacterial profiles in FEP patients, with a particular focus on remission-related subgroups pre- and post-antipsychotic treatment. Our results provide new evidence of distinct microbial compositional and functional signatures in R+ versus R− patients, suggesting that the blood microbiome may serve as a potential tool for predicting treatment response in FEP. By additionally exploring correlations between microbial features and serum cytokine levels, we contribute to a growing understanding of host–microbiome interactions and systemic immune responses in psychosis. Nevertheless, larger-scale studies integrating extended clinical phenotyping, the inclusion of both male and female participants, and complementary experimental variables are necessary to develop robust microbiome–host biomarker signatures for precision diagnostics, monitoring, and treatment in early-stage psychotic disorders.

## Data Availability

The datasets generated and analyzed for this study can be found in the Sequence Read Archive of the National Library of Medicine under BioProject number PRJNA1181866 (https://www.ncbi.nlm.nih.gov/sra/PRJNA1181866, access date: 11 August 2025).

## Supplementary Materials

The following supporting information can be downloaded at https://www.mdpi.com/article/10.3390/microorganisms13081935/s1: Supplementary Figure S1: Rarefaction curves; Supplementary Figure S2: Additional alpha diversity metrics; Supplementary Figure S3: qRT-PCR of 16S rRNA for validation of *Lactococcus*; Supplementary Figure S4: Serum cytokine levels before (t_0_) and after (t_1_) treatment; Supplementary Table S1: Serum cytokine levels in FEP patients; Supplementary Table S2: Pearson correlation between serum cytokine levels and blood microbiome alpha diversity; Supplementary File S1: ASV sequences from blood samples in excel format; Supplementary File S2: Excel file with results from differential abundance analysis of metabolic pathways.

## Abbreviations

The following abbreviations are used in this manuscript:

ASV	Amplicon Sequence Variant
BCAA	Branched-Chain Amino Acid
BMI	Body Mass Index
FC	Fold Change
FDR	False Discovery Rate
FEP	First-Episode Psychosis
NMDS	Non-metric Multidimensional Scaling
NTC	No Template Control
PANSS	Positive and Negative Syndrome Scale
qRT-PCR	quantitative Realt-Time PCR
SCZ	Schizophrenia
