# Supplementary material for: Blood Microbiome Analysis Reveals Biomarkers of Treatment Response in Drug-Naïve Patients with First-Episode Psychosis: A Pilot Study
Source: Microorganisms. 2025 Aug 19;13(8):1935. doi: 10.3390/microorganisms13081935 (PMC12388318; doi:10.3390/microorganisms13081935)
Supplement: Supplementary file 1 [file microorganisms-13-01935-s001.zip › microorganisms-3764382-supplementary/microorganisms-3764382-supplementary/microorganisms-3764382-supplementary-proofed.pdf]

Supplementary Figure S1

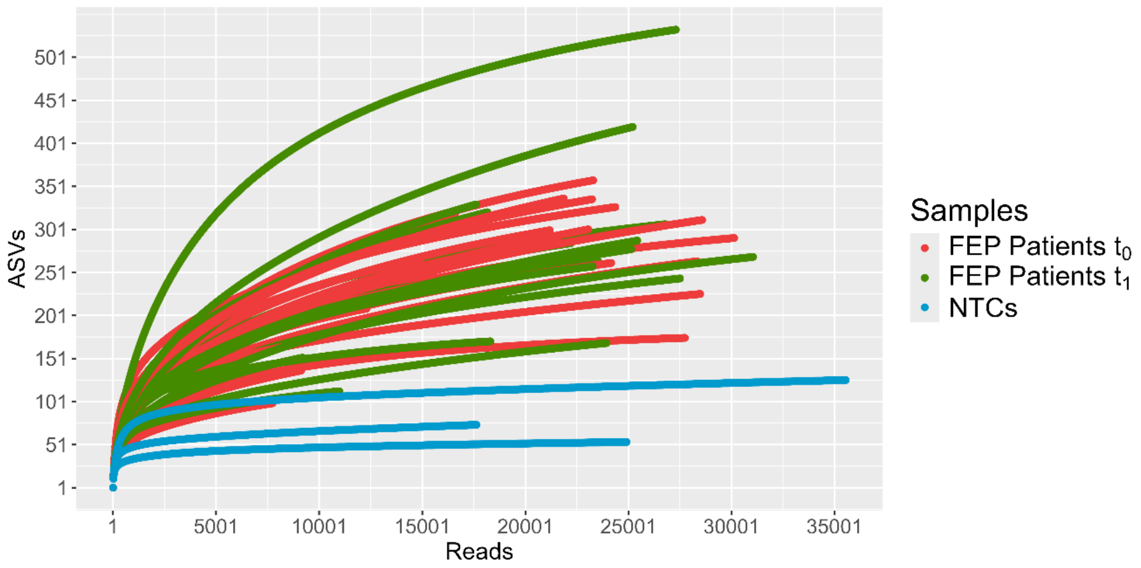

Supplementary Figure S1. Rarefaction analysis of the samples, illustrating the number of observed taxa as a function of sequencing depth. The rarefaction curves indicate that all samples included in the study were sequenced to a sufficient depth to capture the assigned Amplicon Sequence Variants (ASVs).

Supplementary Figure S2

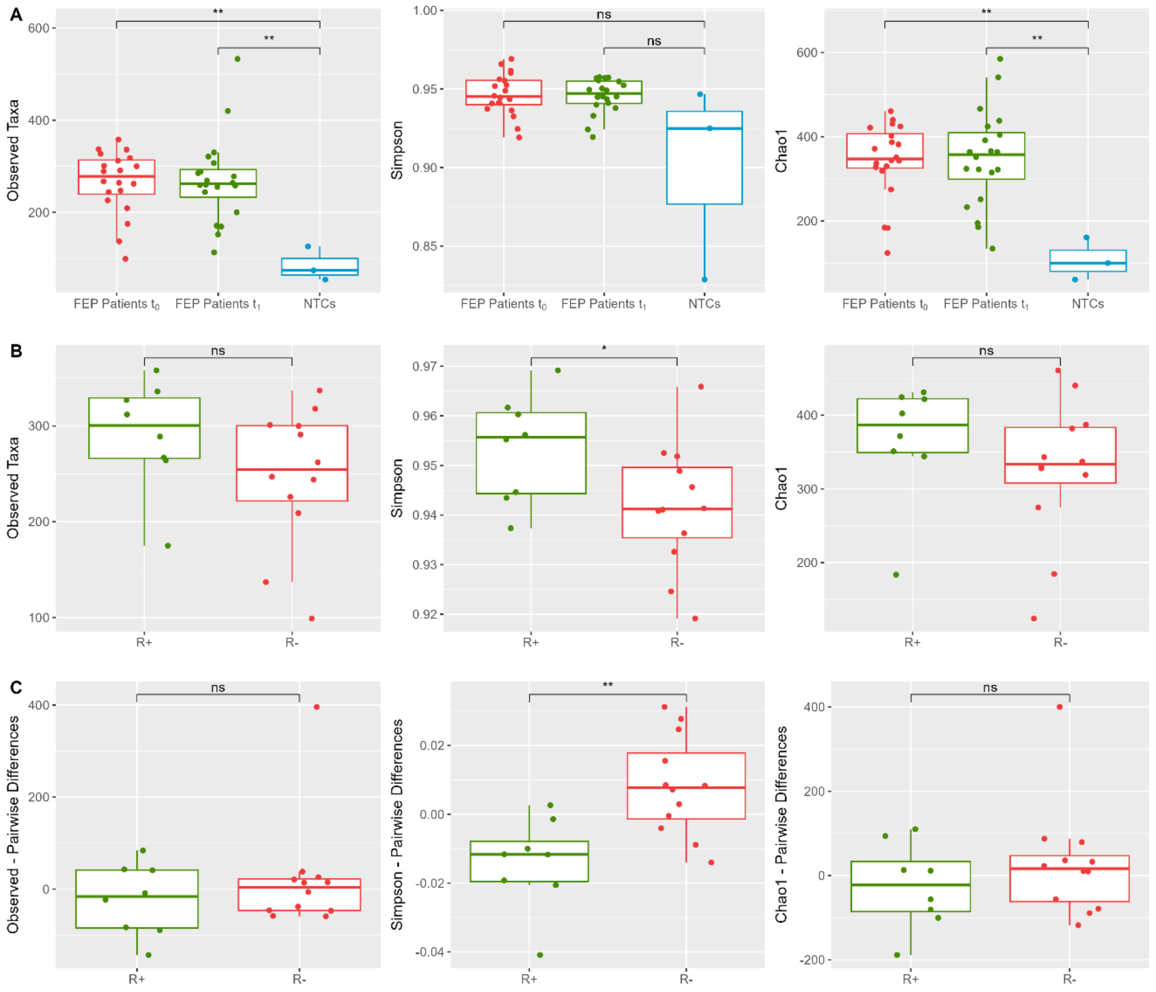

Supplementary Figure S2. (A) Comparison of alpha diversity indices—Observed Taxa, Simpson, and Chao1—in the blood microbiome of FEP patients before (t_0) and after (t_1) antipsychotic treatment, versus Non-Template Control (NTC) samples. Each dot represents an individual sample. (B) Alpha diversity indices (Observed Taxa, Simpson, and Chao1) at baseline in FEP patients stratified by remission status: Remitters (R^+) and Non-Remitters (R^-). (C) Effect of antipsychotic treatment on alpha diversity in FEP patients. Box plots show pairwise differences in alpha diversity indices (Observed Taxa, Simpson, and Chao1) between t_0 and t_1 for each individual. Statistical significance: $p < 0.05$; $p < 0.01$ (Wilcoxon test); ns: not significant. R^+: Remitters; R^-: Non-Remitters.

Supplementary Figure S3

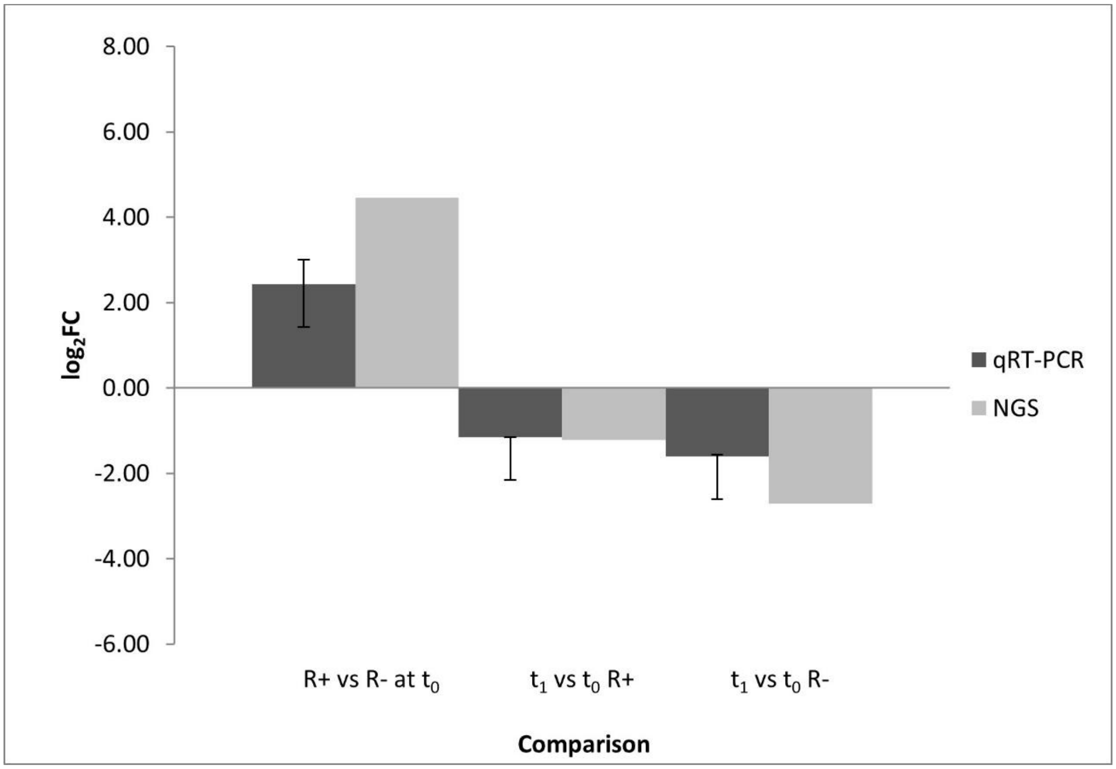

Supplementary Figure S3. Quantitative real-time PCR (qRT-PCR) analysis of 16S rRNA gene amplicons to validate the differential abundance of *Lactococcus raffinolactis* observed in NGS data. Representative patient samples were analyzed and compared as follows: R^+ versus R^- at baseline (t_0; n = 4), and post-treatment (t_1) versus baseline (t_0) within both R^+ and R^- groups (n = 3). Values were normalized to the total bacterial load per sample, as determined by parallel reactions using universal bacterial primers.

Supplementary Figure S4

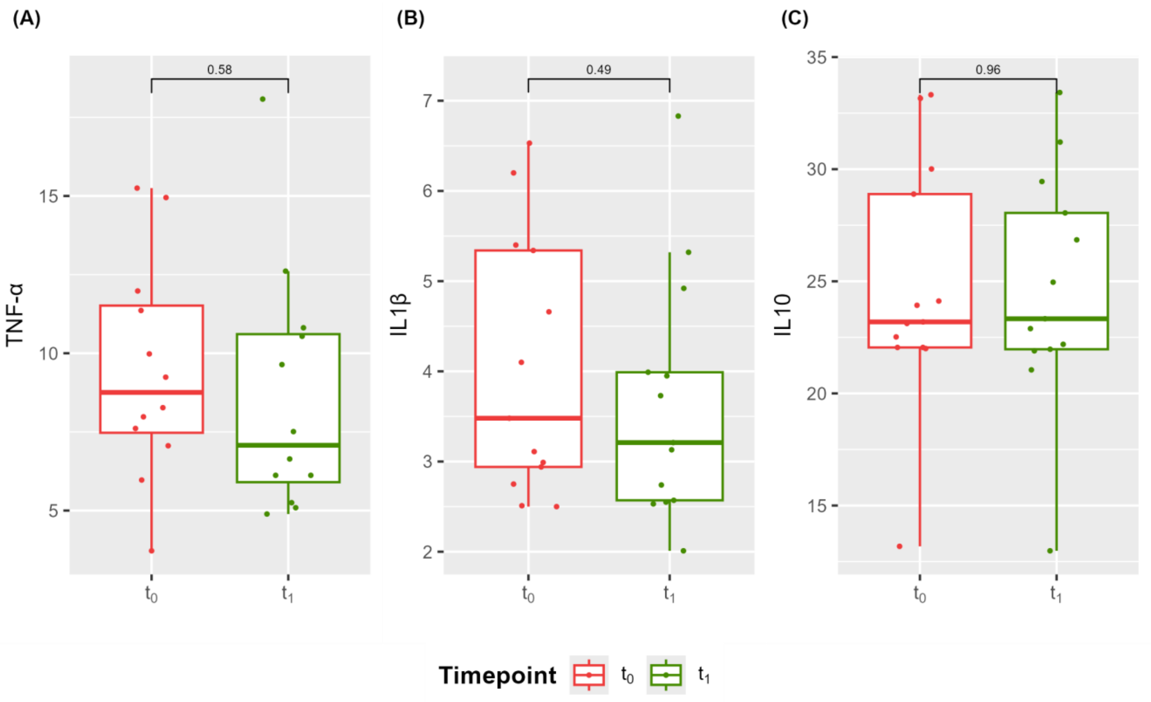

Supplementary Figure S4. Serum levels of TNF-*α* (A), IL1β (B), and IL10 (C) in FEP patients before (t_0) and after (t_1) antipsychotic treatment. Comparisons of cytokine levels between Remitters (R^+) and Non-Remitters (R^-) at each time point were performed using Student's t-test.

Supplementary Table S1

Supplementary Table S1. Serum levels (pg/ml) of TNF-α, IL1β, and IL10 cytokines in FEP patients

Patient number	TNF-α		IL1β		IL10	
	t_0	t_1	t_0	t_1	t_0	t_1
1	*NA**	*NA*	4.66	3.99	*NA*	*NA*
2	7.98**	6.12	*NA*	*NA*	30.01	29.45
3	9.98	7.51	3.48	3.21	33.32	31.21
4	*NA*	*NA*	*NA*	*NA*	*NA*	*NA*
5	3.72	5.25	2.75	2.57	22.05	21.90
6	9.24	18.08	5.40	3.95	13.18	12.98
7	5.97	4.89	2.50	2.74	22.52	22.89
8	11.36	10.54	4.10	2.53	22.05	21.97
9	*NA*	8.35	5.34	4.92	23.12	21.05
10	11.98	10.81	6.20	5.32	28.89	28.05
11	7.06	5.09	3.11	2.55	22.00	22.19
12	8.27	6.12	2.94	2.01	23.19	23.33
13	*NA*	*NA*	*NA*	*NA*	*NA*	*NA*
14	*NA*	*NA*	*NA*	*NA*	*NA*	*NA*
15	15.25	9.64	6.53	6.83	33.16	33.42
16	14.95	12.61	2.99	3.73	24.12	24.96
17	7.61	6.64	2.51	3.13	23.93	26.85
18	*NA*	*NA*	*NA*	*NA*	*NA*	*NA*
19	*NA*	*NA*	*NA*	*NA*	*NA*	*NA*
20	*NA*	*NA*	*NA*	*NA*	*NA*	*NA*
Mean	9.45	8.59	4.04	3.65	24.73	24.63
SD	3.46	3.77	1.43	1.36	5.45	5.28

**NA*: Not Assigned

**Values are log-transformed according to the ELISA report.

t_0: baseline-time of admission

t_1: four weeks from admission

Supplementary Table S2

Supplementary Table S2. Pearson correlation analysis between serum cytokine levels and blood microbiome alpha diversity in FEP patients

Cytokine	Alpha-diversity[a]	Pearson Ro	p-value
TNF-α	t_0[b]	0.01	0.984
	t_1[c]	0.27	0.371
	Pairwise Difference t_1 vs t_0	0.11	0.727
IL1β	t_0	-0.10	0.747
	t_1	-0.14	0.656
	Pairwise Difference t_1 vs t_0	-0.45	0.118
IL10	t_0	-0.28	0.354
	t_1	-0.33	0.277
	Pairwise Difference t_1 vs t_0	-0.59	0.033

[a] Shannon index

[b] t_0: baseline-time of admission

[c] t_1: four weeks from admission
